# Radiological Imaging for Assessing the Respectability of Hilar Cholangiocarcinoma: A Systematic Review and Meta-Analysis

**DOI:** 10.1155/2015/497942

**Published:** 2015-09-01

**Authors:** Hongchen Zhang, Jian Zhu, Fayong Ke, Mingzhe Weng, Xiangsong Wu, Maolan Li, Zhiwei Quan, Yingbin Liu, Yong Zhang, Wei Gong

**Affiliations:** Department of General Surgery, Xinhua Hospital, School of Medicine, Shanghai Jiao Tong University, 1665 Kongjiang Road, Shanghai 200092, China

## Abstract

Hilar cholangiocarcinoma (HCC) remains one of the most difficult tumors to stage and treat. The aim of the study was to assess the diagnostic efficiency of computed tomography (CT), magnetic resonance imaging (MRI), and positron emission tomography/computer tomography (PET/CT) in evaluating the resectability of HCC. A systematic search was performed of the PubMed, EMBASE, and Cochrane databases. Sensitivity, specificity, positive predictive value (PPV), negative predictive value (NPV), and diagnostic accuracy were calculated for individual studies and pooled data as well as test for heterogeneity and public bias. Our data showed that CT had the highest pooled sensitivity at 95% (95% CI: 91–97), whereas PET/CT had the highest pooled specificity at 81% (95% CI: 69–90). The area under the curve (AUC) of CT, MRI, and PET/CT was 0.9269, 0.9194, and 0.9218, respectively. In conclusion, CT is the most frequently used imaging modality to assess HCC resectability with a good sensitivity and specificity. MRI was generally comparable with that of CT and can be used as an alternative imaging technique. PET/CT appears to be the best technique in detecting lymph node and distant metastasis in HCC but has no clear role in helping to evaluate issues of local resectability.

## 1. Introduction

Hilar cholangiocarcinoma (HCC), a rare malignant tumor arising from the epithelium of the bile ducts, is usually encountered at an advanced stage accordingly with a poor prognosis [1]. Surgery is still the only potentially curative treatment for HCC. However, surgical strategies should be made and tailored based on the location, the size, and the vascular invasion of the tumors [2]. Consequently, accuracy of preoperative evaluation and staging are critical to sort out potentially resectable cases for radical resection and to avoid unnecessary surgical interventions for those unresectable cases. The latter circumstances generally include liver metastasis, distant lymph node metastasis, bilateral arterial or portal invasion, unilateral vascular invasion, and contralateral lobar atrophy and distant metastases [3] as follows.

The criteria for unresectability of hilar cholangiocarcinoma are as follows:

 patient factor:
   medically unfit/unable to tolerate a major operation,
 hepatic cirrhosis local tumor-related factor:
   tumor extension to secondary biliary radicles bilaterally,
   encasement or occlusion of the main portal vein proximal to its bifurcation,
   atrophy of one hepatic lobe with contralateral portal vein branch encasement or occlusion,
   atrophy of one hepatic lobe with contralateral tumor extension to secondary biliary radicles,
   unilateral tumor extension to secondary biliary radicles with contralateral portal vein branch encasement or occlusion,
  metastatic disease:
   histologically proven metastases to N2 lymph nodes,
   lung, liver, or peritoneal metastases.

The breadth and complexity of disease progressions challenge preoperative evaluation.

Computed tomography (CT), magnetic resonance imaging (MRI), and positron emission tomography/computer tomography (PET/CT) are generally used to demonstrate the primary lesion and staging of HCC [4]. The links between the performance of imaging modalities and the judgement on the resectability of HCC have not been systematically reviewed. We meta-analyzed the current researches on CT, MRI, and PET/CT to view the role of the structural and functional imaging in assessing the resectability of HCC, which may benefit further studies.

## 2. Materials and Methods

### 2.1. Search Strategy

The databases of MEDLINE, EMBASE, CancerLit, and the Cochrane Library were searched from January 1980 to March 2015 using the following key words with the appropriate combinations: “CT” or “computed tomography,” “Magnetic Resonance Imaging” or “MRI,” “positron emission tomography/computer tomography” or “PET/CT,” “hilar cholangiocarcinoma,” “Klatskin tumour,” “Bile Duct Neoplasms,” “resectability,” and “diagnosis.” The search was limited to “human only” and “English language only.” All review articles, letters, comments, and case reports were eliminated. Articles found to be eligible on the basis of their title and abstract were subsequently selected for full manuscript review. We augmented our literature search by manually reviewing the reference lists of identified studies. Any differences were resolved by mutual agreement.

### 2.2. Inclusion Criteria

The following inclusion criteria were applied: (1) articles were published in English; (2) CT, MRI, or PET/CT was used to evaluate the resectability of HCC; (3) for per-patient statistics, sufficient data were presented to calculate the true-positive (TP), false-negative (FN), false-positive (FP), and true-negative (TN) values; (4) 10 or more patients were included; (5) when data or subsets of data were presented in more than one article, the article with the most detail or the most recent article was chosen. Authors of abstracts and studies that did not report sufficient data were contacted to request additional information.

### 2.3. Data Extraction

Two observers independently extracted relevant data by using a standardized form including the following items: first author, year of publication, country, sample size, description of study population (age), study design (prospective, retrospective, or unknown), and patient enrollment (consecutive or not). The results of TP, FN, FP, and TN in the evaluation of the resectability of HCC were extracted on a per-patient basis. Sensitivity was calculated by determining the percentage of patients predicted to be resectable in the group of patients who were resectable. Specificity was determined by the percentage of patients predicted to be unresectable in the group of patients who were unresectable. Disagreement was resolved by means of consensus.

### 2.4. Quality Assessment

Methodological quality of the selected studies was assessed by the Quality Assessment of Diagnostic Accuracy Studies (QUADAS) tool. This evidence-based tool includes 14 quality items, each of which was phrased as a question and scored as “yes,” “no,” or “unclear.” The “yes” score ranged from 0 to 14. Fulfillment of the methodological quality criteria for each article was considered high, acceptable, or low when the “yes” score covered >70%, 50–70%, or <50%, respectively. A more detailed description of each item and a guide to score each item were provided by Whiting et al. in 2003 [5]. The study design of prospective or retrospective was also recorded.

### 2.5. Statistical Analysis

The statistical software Meta-Disc (version 1.40) and STATA (version 12.0; Stata Corp.) were used to analyze the data of CT, MRI, and PET/CT. The pooled sensitivity, specificity, diagnostic odds ratio (DOR), summary receiver operating characteristic curves (SROC), and the ^*∗*^ *Q* index were calculated for each modality. The ^*∗*^ *Q* index was defined by the point at which the sensitivity and specificity were equal, which was closest to the ideal top-left corner of the SROC space [6]. The *Z*-test was then performed to determine statistical significance of the sensitivity, specificity, DOR, and SROC of each modality from the other two. Heterogeneity was tested with the Higgins and Thompson test, and the *I* ^2^ statistic was calculated ranging from 0% (no heterogeneity) to 100% (all variance due to heterogeneity). In contrast to the Cochran-*Q*, the *I* ^2^ is less affected by the number of studies included in a meta-analysis [7]. If no or moderate heterogeneity is found (*I* ^2^ ≤ 50%), pooling is justified. A fixed effects model (FEM) was used when homogeneity existed among different studies, whereas a random effects model (REM) was used when heterogeneity was found. In addition, the presence of publication bias was visually assessed by producing a funnel plot.

## 3. Results

### 3.1. Study Identification and Eligibility

**Figure 1:**
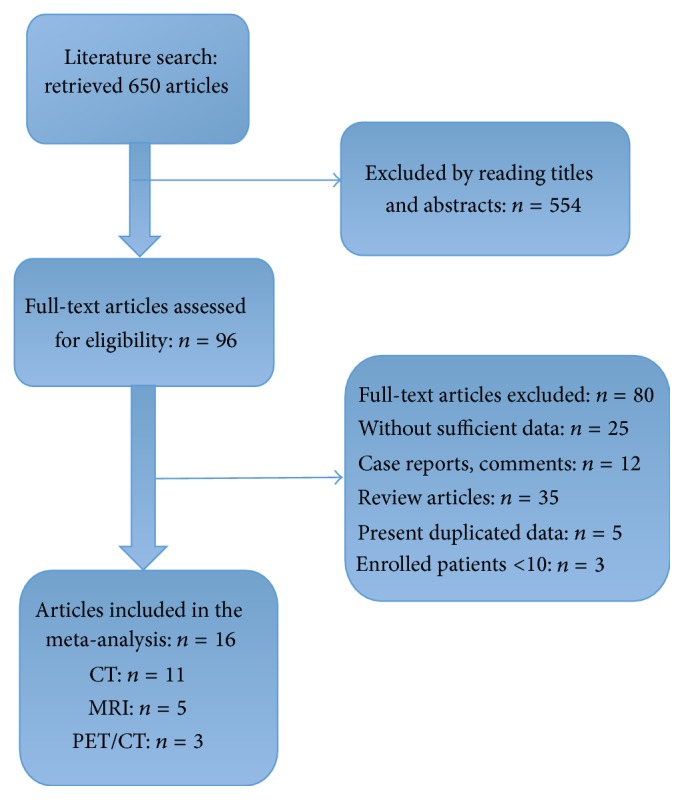
Flow chart and study selection.

The results of the literature search and study selection are shown in Figure 1. According to the search strategy, 650 articles were initially retrieved and 554 were excluded based on review of the title and abstract. Another 80 did not meet the inclusion criteria and were excluded later. Finally, a total of 16 studies including 651 patients were eligible for the meta-analysis, of which 11 were CT studies [8–18], 5 were MRI studies [13, 14, 19–21], and 3 were PET/CT studies [14, 22, 23].

### 3.2. Quality Assessment

The quality assessment scores of 16 studies showed high quality ranging from 10 to 12, with a mean study quality score of 11. The imaging findings were probably known during surgery and therefore the reference standard was generally not blinded to the results of the index test (QUADAS item 11). The time period between imaging and the reference standard was mentioned in only 7 studies [10, 13, 15, 19–21, 23] and was 14 days or less in 6 studies [13, 15, 19–21, 23]. Inclusion and exclusion criteria were clearly mentioned in all studies.

### 3.3. Study Characteristics

The characteristics of the included studies are presented in Table 1. These studies included a total of 651 eligible patients (median 41 patients). Reported age ranged from 21 to 88 years, and the proportion of male patients was 46% to 87%. 11 articles studied the performance of CT [8–12, 15–18], 3 MRI [19–21], and 2 PET/CT [22, 23]. One small study [13] including 27 patients compared two investigations (CT and MRI) head to head. One study [14] including 123 patients assessed the three modalities in the same patient population. Four studies [10, 14, 15, 22] were prospective and 8 [8, 9, 12, 13, 17, 20, 21, 23] were retrospective, and the design of the other four studies [11, 16, 18, 19] was unclear. In 9 studies [8, 10–12, 14, 15, 17, 19, 21] patients were enrolled in a consecutive manner. The TP, FN, FP, and TN results and diagnostic performance of imaging in each study are shown in Tables 2 [Table tab3]–4.

### 3.4. Publication Bias and Heterogeneity

Deeks' funnel plots are shown in Figures 8 [Fig fig9]–10. The results did not suggest a publication bias (*p*, CT = 0.57; *p*, MRI = 0.13; and *p*, PET/CT = 0.39).

No significant heterogeneity of diagnostic performance was found for the CT and MRI studies (sensitivity: *I* ^2^ = 40.5% and 47.8%, resp.; specificity: *I* ^2^ = 33.1% and 0%, resp.), while the specificity of the PET/CT studies (specificity: *I* ^2^ = 52.4%; sensitivity: *I* ^2^ = 0%) showed a moderate heterogeneity. Therefore, a fixed effects model was chosen for the CT and MRI studies, and random effects model was for PET/CT studies.

### 3.5. Summary Estimates of the Sensitivity, Specificity, and Diagnostic Odds Ratio

**Figure 2:**
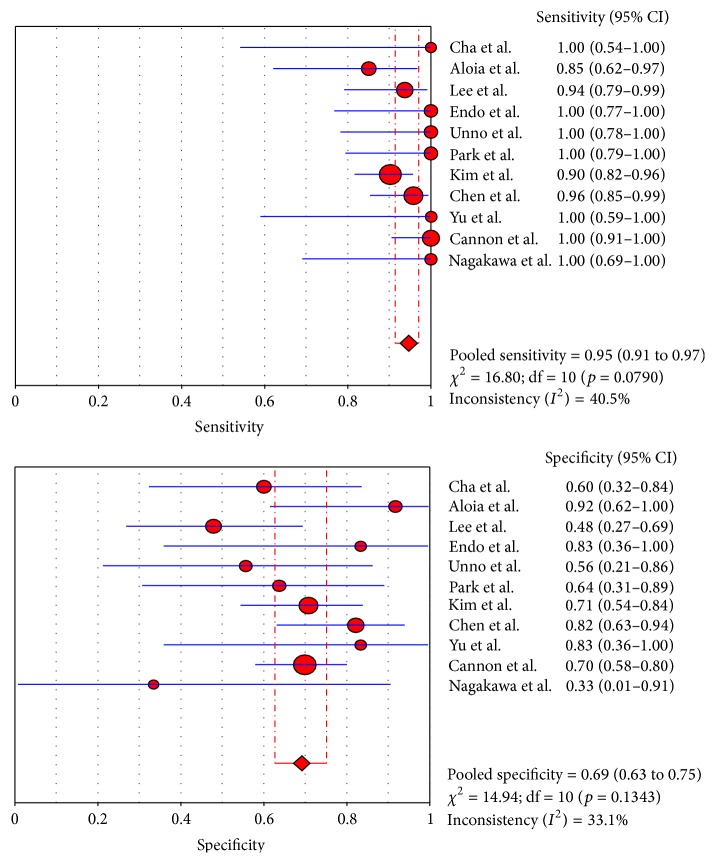
Sensitivity and specificity of CT.

**Figure 3 fig3:**
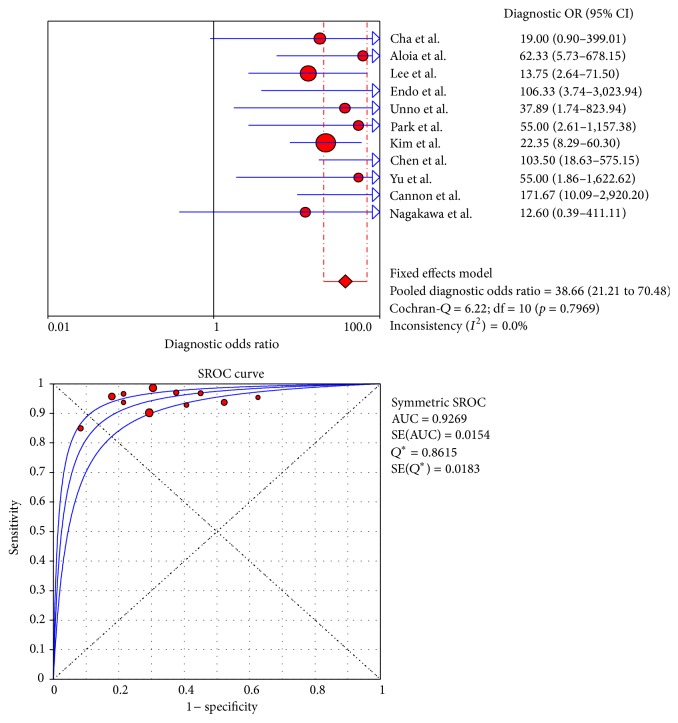
DOR and SROC curve of CT.

**Figure 4 fig4:**
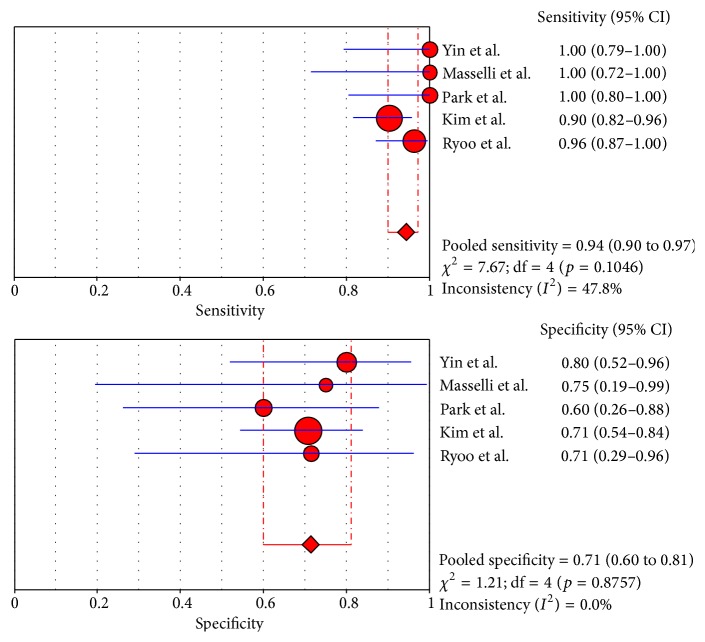
Sensitivity and specificity of MRI.

**Figure 5 fig5:**
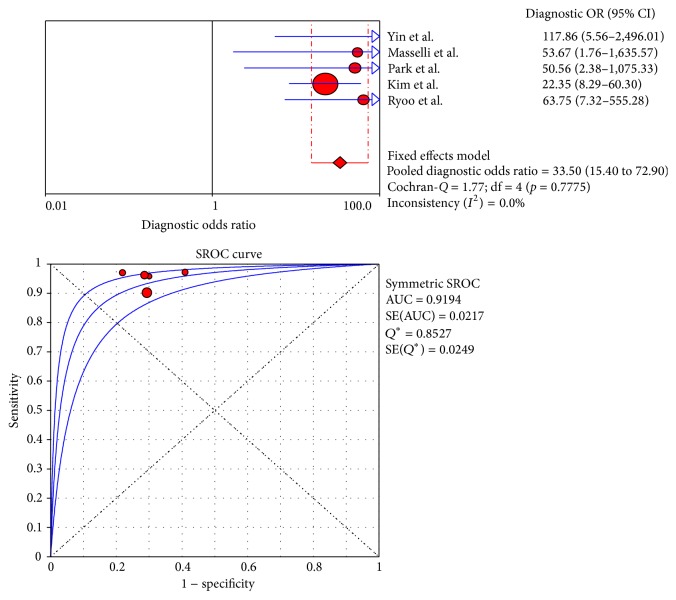
DOR and SROC curve of MRI.

**Figure 6 fig6:**
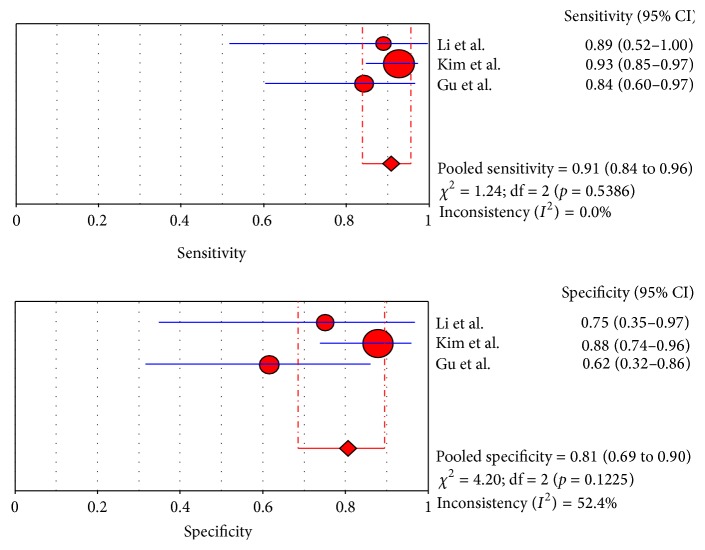
Sensitivity and specificity of PET/CT.

**Figure 7:**
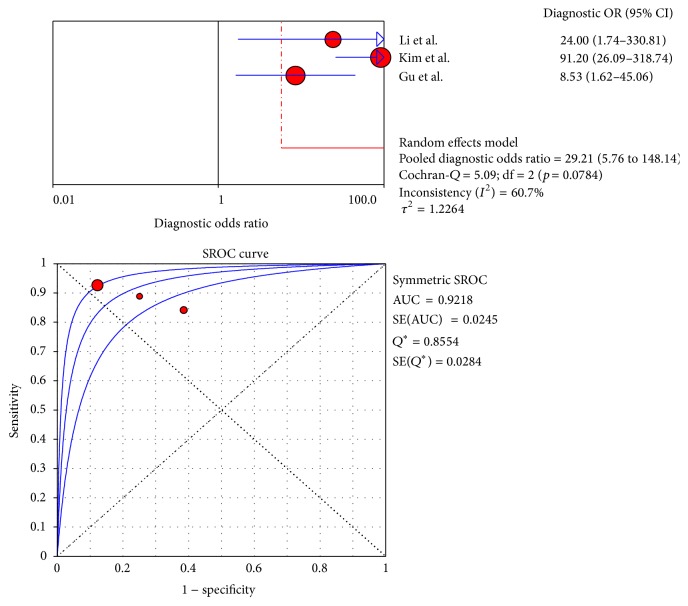
DOR and SROC curve of PET/CT.

**Figure 8:**
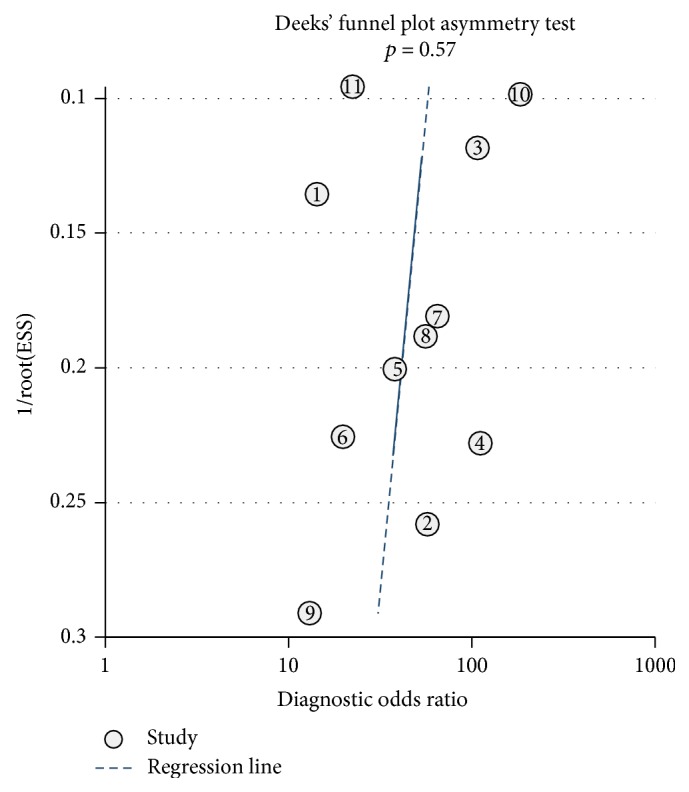
Deeks' funnel plots for assessing the publication bias risk of CT.

**Figure 9 fig9:**
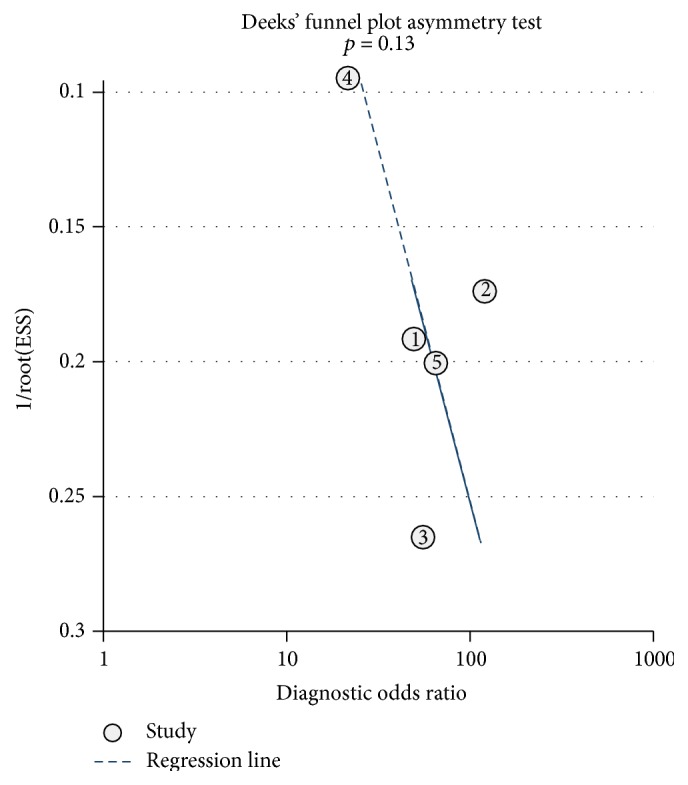
Deeks' funnel plots for assessing the publication bias risk of MRI.

**Figure 10:**
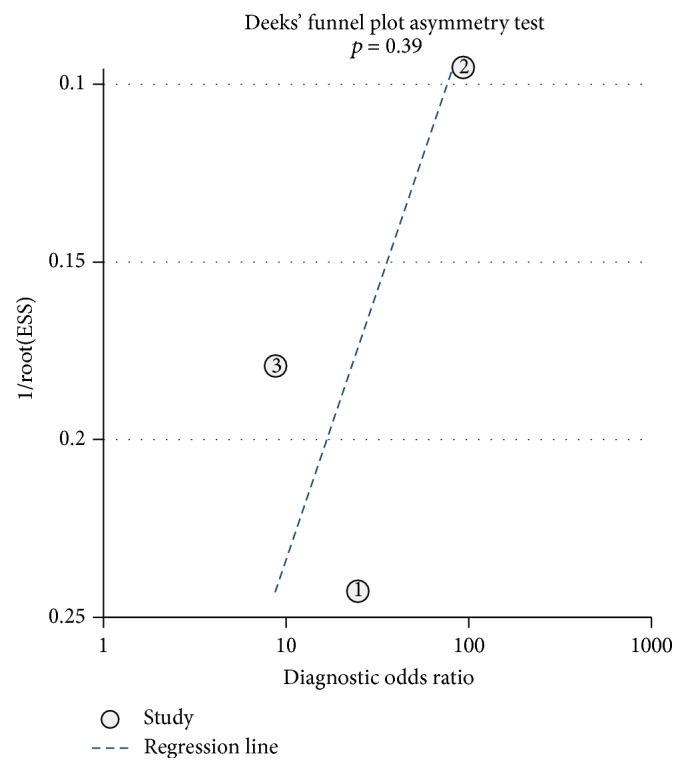
Deeks' funnel plots for assessing the publication bias risk of PET/CT.

The pooled sensitivities for CT, MRI, and PET/CT were 95% (95% CI: 91–97), 94% (95% CI: 90–97), and 91% (95% CI: 84–96), respectively. Although CT and MRI had a higher pooled sensitivity than PET/CT (*p* < 0.05), no statistically significant difference was found between CT and MRI (*p* > 0.05). The pooled specificities for CT, MRI, and PET/CT were 69% (95% CI: 63–75), 71% (95% CI: 60–81), and 81% (95% CI: 69–90), respectively, and no statistically significant difference was found among the three modalities (*p* > 0.05). The forest plots for the sensitivities and specificities are shown in Figures 2 [Fig fig3] [Fig fig4] [Fig fig5] [Fig fig6]–7. The pooled DOR was 38.66 (95% CI: 21.21–70.48) for CT with the heterogeneity *I* ^2^ at 0.0% (*p* = 0.7969) and 33.50 (95% CI: 15.40–72.90) for MRI with the heterogeneity *I* ^2^ at 0.0% (*p* = 0.7775). Meanwhile, the DOR for PET/CT was 35.01 (95% CI: 14.24–86.05), with the heterogeneity *I* ^2^ at 60.7% (*p* = 0.0784) (Table 5).

### 3.6. Summary of the Receiver Operating Characteristic Curves and Area under the Curve

The SROC curves for CT, MRI, and PET/CT are shown in Figures 3, 5, and 7. Given the heterogeneity, REM was used to synthesize the ROC curves for PET/CT, whereas FEM was used for CT and MRI. The AUC values of CT, MRI, and PET/CT were 0.9269, 0.9194, and 0.9218, respectively (Table 5). No significant difference was found among the three imaging modalities (*p* > 0.05).

## 4. Discussion

Hilar cholangiocarcinoma (HCC) remains one of the most difficult tumors to stage and treat [24]. Resectability of HCC patients is defined by factors that are important when considering patients for any major liver resection, such as physical condition, age, and size and function of the future remnant liver. In addition, resectability is defined by factors specific for HCC, including invasion of the portal vein and hepatic artery, lymph node status, and proximal ingrowth into the segmental bile ducts [25]. These specific factors can be assessed preoperatively with acceptable accuracy. Although imaging modalities cannot become the determinants of resectability in patients with HCC, accurate preoperative assessment of tumor extent and resectability with an appropriate imaging study is one of the most important steps in treatment planning [26].

### 4.1. CT

Because of its wide availability, CT is the most frequently used imaging modality to assess the resectability of biliary tumors. High resolution CT allows for accurate depiction of a thickened bile duct wall and tumor spread into liver parenchyma or hilar vessels [10]. Three-dimensional (3D) imaging has been commonly used for the evaluation of biliary tract anatomy. In addition, multiplanar reconstruction (MPR) imaging has been used to assess vertical spread, such as tumor invasion of the major vessel, and horizontal spread of the tumor in the bile duct [11]. One systematic review of HCC imaging techniques demonstrated an acceptable accuracy (86%) of CT in assessing ductal extent of HCC [27] and showed that the sensitivity and specificity estimates of CT were 89% and 92% for evaluation of portal vein involvement and 83% and 93% for hepatic artery involvement, respectively. However, it has been reported that CT may underestimate lymph node involvement and peritoneal metastases. In the series by Cha et al. [8], among 21 patients with HCC, CT correctly detected the unresectable tumor in 9 cases with a 100% NPV. However, in 12 patients with suspected resectable disease by CT, 6 cases turned out to be unresectable (PPV: 50%). Besides, CT failed to detect small hepatic metastasis (*n* = 1), lymph node metastasis (*n* = 1), extensive tumor (*n* = 2), and variation of bile duct (*n* = 2), which precluded surgical resection.

This meta-analysis of CT, MRI, and PET/CT revealed that CT with the highest pooled sensitivity of 95% was able to accurately estimate the resectability of HCC. Nevertheless, CT is limited in detecting small hepatic or lymph node metastasis. Tumor spread to normal-sized lymph nodes and hepatic metastases smaller than 1 cm size seem to be beyond the power of current imaging techniques [28].

### 4.2. MRI

More recently, MRI in conjunction with magnetic resonance cholangiopancreatography (MRCP) has proven to be helpful in diagnosing HCC and assessing resectability [29]. It can provide an accurate map of the biliary tree even in the undrained segment and demonstrates extra ductal tumors directly and noninvasively. Furthermore, MRI and MRCP can visualize the different components: bile ducts, vessels, and invasion of adjacent liver parenchyma [30]. However, pictures of vascular invasion by MRI are still inferior to those of MDCT, and evaluation of lymph node metastasis is less feasible on MRI because of the low spatial resolution of the technique [13]. In the study by Masselli et al. [20] MRI correctly predicted vascular involvement in 73% and liver involvement in 80% of the cases. The number of overall correctly assessed patients with regard to resectability was 11 true positive, 1 false positive, and 3 true negative. In a comparison of MRI/MRCP versus MDCT with direct cholangiography, Park and colleagues [13] demonstrated no difference between the two groups in assessing HCC resectability. The overall accuracy was 77.8% for both MRI and MRCP.

Our meta-analysis showed that MRI had a pooled sensitivity of 94% and specificity of 71%, generally comparable to CT in the evaluation of the resectability of HCC. Therefore, MRI/MRCP can be used as an alternative imaging technique to establish the diagnosis of HCC due to the additional benefits of having a shorter preoperative diagnostic time, being a noninvasive procedure, and not exposing patients to radiation [31].

### 4.3. PET/CT

Since malignant tumor cells often show increased glucose metabolism, whole body PET/CT imaging with the tracer fluoro-2-deoxy-D-glucose (FDG) has been used as a functional imaging to evaluate the metastasis of HCC [32]. PET/CT, which combines a full-ring detector clinical PET scanner and multidetector computed tomography (MDCT) scanner, acquires both metabolic and anatomic imaging data with a single device during a single diagnostic session. It has the advantage of surveying the entire body and becomes more valuable than CT and MRI in detecting lymph node and distant metastases. Unnecessary surgery can be avoided if patients with advanced disease are defined by PET/CT [33]. For 123 patients with suspected cholangiocarcinoma enrolled in Kim et al.'s study [14], the overall values for sensitivity, specificity, and accuracy of PET/CT in primary tumor detection were 84.0%, 79.3%, and 82.9%, respectively. PET/CT demonstrated no statistically significant advantage over CT and MRI/MRCP in the diagnosis of primary tumor. However, PET/CT revealed significantly higher accuracy than CT and MRI in the diagnosis of regional lymph nodes metastases (75.9% versus 60.9%, *p* = 0.004) and distant metastases (88.3% versus 78.7%, *p* = 0.004). Additionally, PET/CT corrected resectability in 15 (15.9%) cases of cholangiocarcinoma who had been falsely recognized by CT and MRI. In those 15 patients, the stage was upgraded from resectable to unresectable in 7 and downgraded from unresectable to resectable in 8. In our study, PET/CT showed the highest pooled specificity of 81%, compared to 71% for MRI and 69% for CT. A high specificity assures surgeons resectability determined by the findings of PET/CT with a great deal of certainty. A 91% pooled sensitivity of PET/CT also indicated that PET/CT had a good power to define the settings of unresectability. Nevertheless, the small number of patients included in some studies of this meta-analysis may have influence on the results. Although this molecular imaging technique is becoming increasingly available, PET/CT remains an expensive imaging tool which necessitates ionizing radiation exposure. Furthermore, FDG is not a tumor-specific substance. Increased FDG accumulation may be observed in a variety of benign entities and in some physiologic conditions, which may yield false-positive findings, and the limited spatial resolution may also reduce the accuracy of the technique. More studies on the application of PET/CTs are needed to further investigate the benefits of this imaging modality in HCC.

This could be the first meta-analysis in assessing the performance of imaging modalities in the evaluation of the resectability/unresectability of HCC. Several limitations need to be considered. The heterogeneity between the included studies may indicate the potential bias for the meta-analysis. Due to the possibility of publication bias, the imaging characteristics varied among studies, such as the type of CT and the relevant parameters. The conclusion on MRI and PET/CT may be biased by the low number of datasets. Ideally, the diagnostic efficiency of different imaging measures should be assessed in the same patient population. The pros and cons of diagnostic imaging can be more easily and accurately defined and compared. In our investigation we only found two such studies and more researches comparing imaging efficiency on the same study population are expected.

## 5. Conclusion

In summary, CT is the most frequently used imaging modality to assess HCC resectability with a good sensitivity and specificity. MRI was generally comparable with that of CT and can be used as an alternative imaging technique. PET/CT appears to be the best technique in detecting lymph node and distant metastasis in HCC but has no clear role in helping to evaluate issues of local resectability. Future researches with a bigger sample size, a more reasonable design, are required to yield more efficient diagnostic strategy. Furthermore, cost-effectiveness analyses of MRI and PET/CT in patients with HCC are also needed.

In clinical settings, CT, MRI, and PET/CT are used nowadays either alone or in various combinations with each other for assessing resectability of HCC. The information provided by CT, MRI, or PET/CT is often complementary because these methods are based on different biophysical foundations. Therefore, combining diagnostic information from these modalities can add diagnostic certainty and also prove beneficial for an optimized and individualized treatment plan.

## Figures and Tables

**Table 1:** Main characteristics of the included studies.

Author	Year of publication	Country	Number of patients	Age, mean (range)	Patients selection	Modality	Study design	QUADAS Score
Cha et al. [8]	2000	Korea	21	56 (34–69)	Consecutive	CT	Retrospective	11
Lee et al. [9]	2006	Korea	55	59 (29–76)	NA	CT	Retrospective	12
Aloia et al. [10]	2007	USA	32	67	Consecutive	CT	Prospective	11
Endo et al. [11]	2007	Japan	20	65 (50–80)	Consecutive	CT	NA	12
Unno et al. [12]	2007	Japan	24	64	Consecutive	CT	Retrospective	11
Yin et al. [19]	2007	China	31	53 (21–74)	Consecutive	MRI	NA	12
Masselli et al. [20]	2008	Italy	15	58 (49–74)	NA	MRI	Retrospective	12
Park et al. [13]	2008	Korea	27	60 (43–80)	NA	CT MRI	Retrospective	12
Li et al. [22]	2008	Germany	17	62	NA	PET/CT	Prospective	11
Kim et al. [14]	2008	Korea	123	60 (28–78)	Consecutive	CT MRI PET/CT	Prospective	12
Chen et al. [15]	2009	China	75	60	Consecutive	CT	Prospective	12
Yu et al. [16]	2010	China	13	65 (54–79)	NA	CT	NA	11
Ryoo et al. [21]	2010	Korea	60	66 (45–77)	Consecutive	MRI	Retrospective	12
Cannon et al. [17]	2012	USA	110	64 (21–88)	Consecutive	CT	Retrospective	11
Gu et al. [23]	2012	China	32	56	NA	PET/CT	Retrospective	10
Nagakawa et al. [18]	2014	Japan	13	65 (39–83)	NA	CT	NA	11

NA = data not available.

**Table 2:** TP, FP, FN, TN, and diagnostic performance of CT.

Author	TP	FP	FN	TN	Sensitivity (%)	Specificity (%)	PPV (%)	NPV (%)	Accuracy (%)
Cha et al. [8]	6	6	0	9	100	60	50.0	100	71.4
Lee et al. [9]	30	12	2	11	93.8	47.8	71.4	84.6	74.5
Aloia et al. [10]	17	1	3	11	85	91.7	94.4	78.6	87.5
Endo et al. [11]	14	1	0	5	100	83.3	93.3	100	95
Unno et al. [12]	15	4	0	5	100	55.6	78.9	100	83
Park et al. [13]	16	4	0	7	100	63.6	80.0	100	85
Kim et al. [14]	74	12	8	29	90.2	70.7	86.0	78.4	83.7
Chen et al. [15]	45	5	2	23	95.7	82.1	90.0	92.0	91
Yu et al. [16]	7	1	0	5	100	83.3	87.5	100	92
Cannon et al. [17]	37	22	0	51	100	69.9	62.7	100	80
Nagakawa et al. [18]	10	2	0	1	100	33.3	83.3	100	85

TP: true positive; FP: false positive; TN: true negative; FN: false negative.

**Table 3 tab3:** TP, FP, FN, TN, and diagnostic performance of MRI.

Author	TP	FP	FN	TN	Sensitivity (%)	Specificity (%)	PPV (%)	NPV (%)	Accuracy (%)
Yin et al. [19]	16	3	0	12	100	80	84.2	100	90.3
Masselli et al. [20]	11	1	0	3	100	75	91.7	100	93.3
Park et al. [13]	17	4	0	6	100	60	80.9	100	85
Kim et al. [14]	74	12	8	29	90.2	70.7	86.0	78.4	83.7
Ryoo et al. [21]	51	2	2	5	96.2	71.5	96.2	71.4	93.3

**Table 4:** TP, FP, FN, TN, and diagnostic performance of PET/CT.

Author	TP	FP	FN	TN	Sensitivity (%)	Specificity (%)	PPV (%)	NPV (%)	Accuracy (%)
Li et al. [22]	8	2	1	6	88.9	75	80.0	85.7	82
Kim et al. [14]	76	5	6	36	92.7	87.8	93.8	85.7	91.1
Gu et al. [23]	16	5	3	8	84.2	61.5	76.2	72.7	75

**Table 5:** Summary estimates of sensitivity, specificity, DOR, and AUC for CT, MRI, and PET/CT.

Modality	Pooled Sensitivity (95% CI)	Pooled Specificity (95% CI)	DOR	^*∗*^ *Q*	AUC
CT	95% (91–97%)	69% (63–75%)	38.66 (21.21–70.48)	0.8615	0.9269
MRI	94% (90–97%)	71% (60–81%)	33.50 (15.40–72.90)	0.8527	0.9194
PET/CT	91% (84–96%)	81% (69–90%)	35.01 (14.24–86.05)	0.8554	0.9218
